# P02.10. 10-year experience from an integrative pediatric oncology centre: a retrospective analysis

**DOI:** 10.1186/1472-6882-12-S1-P66

**Published:** 2012-06-12

**Authors:** A Laengler, R Seiler, T Zuzak, T Ostermann, G Kameda, P Heusser

**Affiliations:** 1 Centre for Integrative Pediatric Oncology Gemeinschaftskrankenhaus, Herdecke, Germany; 2 Witten Herdecke University, Witten, Germany; 3 University of Essen Pediatric Oncology, Essen, Germany

## Purpose

Complementary and alternative therapies (CAM) are widely used in pediatric oncology. Till now there are no published data on the clinical practice of integrating CAM into standard pediatric oncology (PO) care.

## Methods

We performed a retrospective analysis of patient records of all PO-patients treated in our integrative pediatric oncology centre (IPOC) with 1st admission between January 1^st^ 1999 and December 31 ^st^ 2008. Inclusion-criteria were: age 0-18 years; proven cancer-diagnosis; and at least one conventional intervention (chemotherapy, operation, radiation) in our hospital.

## Results

A total of 116 cases (39% female) were analysed. Seventy-eight percent were admitted with their 1 ^st^ cancer-diagnosis, 22% with any relapse. Fifty-eight percent received their diagnosis in our hospital, 42% in another hospital (9% of them outside Germany). The spectrum of diagnosis was the same as in the German PO-population. Along with a standard conventional therapy according to actual treatment-plans within the GPOH- and/or SIOP-framework, 91% of our patients received some form of CAM-medication (e.g. 75% mistletoe-preparations), most out of the spectrum of Anthroposophic Medicine (AM). We could identify a Top 10 of AM remedies prescribed to most patients. The most used non-pharmacological CAM-interventions were: 63% eurythmy therapy, 56% art-therapies (painting, sculpturing), 51% music therapy, 46% external embrocations and compresses. There were no reported serious adverse events for any of the pharmacological or non-pharmacological CAM interventions.

## Conclusion

It is possible to integrate CAM into an IPOC within the standard-spectrum of PO-diseases. In a retrospective analysis over a 10-year-period we found no indices for relevant toxicities. Statements regarding the effectiveness of this approach cannot be drawn from this retrospective analysis. Further prospective studies of this approach are necessary.

